# Low complement items play different roles on the classification performance of SLICC-2012, EULAR/ACR-2019, and SLERPI

**DOI:** 10.3389/fmed.2025.1695383

**Published:** 2025-12-12

**Authors:** Shanshan Chen, Lin Zhang, Mengxue Yan, Siping Li, Leixi Xue, Mei Tang

**Affiliations:** 1 Department of Rheumatology and Immunology, The Second Affiliated Hospital of Soochow University, Suzhou, Jiangsu, China; 2 Department of Critical Care Medicine, Lanxi People’s Hospital, Lanxi, Zhejiang, China

**Keywords:** systemic lupus erythematosus, low complement, SLICC-2012, EULAR/ACR-2019, SLERPI

## Abstract

**Objectives:**

This study aimed to explore the association of low complement items with the performance of the systemic lupus erythematosus (SLE) classification criteria.

**Methods:**

This study included 352 patients with SLE and 385 individuals with positive antinuclear antibodies with other diseases. The performance of the Systemic Lupus International Collaborating Clinics (SLICC)-2012, European League Against Rheumatism and American College of Rheumatology (EULAR/ACR)-2019, and SLE Risk Probability Index (SLERPI) were compared before and after excluding the low complement item.

**Results:**

The exclusion of the low complement item decreased the sensitivity and increased the specificity of SLICC-2012 and EULAR/ACR-2019, whereas SLERPI demonstrated stable classification performance in classifying patients with and without SLE. SLICC-2012 exhibited the highest sensitivity in identifying patients with SLE with hypocomplementemia (99.0%; 95% confidence interval [CI]: 97.2–99.8%), followed by SLERPI (98.4%; 95% CI: 96.3–99.5%) and EULAR/ACR-2019 (97.7%; 95% CI: 95.4–99.1%). After excluding the low complement item, the sensitivities decreased to 93.0% (89.5–95.5%) for SLICC-2012, 97.5% (86.8–99.9%) for SLERPI, and 93.3% (90.0–95.8%) for EULAR/ACR-2019. SLICC-2012, EULAR/ACR-2019, and SLERPI identified 16, 17, and 21 patients with SLE with hypocomplementemia before clinical diagnosis, decreasing to 10, 16, and 20 cases after excluding the low complement item. Further, patients with SLE with both low C3 and C4 levels demonstrated shorter disease duration, higher acute cutaneous lupus prevalence, nonscarring alopecia, and increased positivity rates for anti-DNA antibodies, antiphospholipid antibodies, and direct Coombs’ tests compared with those with low C3 or C4 levels. The SLE group with low C3 and C4 levels demonstrated significantly higher total scores than the SLE group with low C3 or C4 levels in all three classification criteria, and this significance persisted even after excluding the low complement item.

**Conclusion:**

The low complement item strongly improved the sensitivity and early classification capacity of SLICC-2012 while minimally influencing SLERPI. Furthermore, higher weights assigned to combined low C3 and C4 levels in EULAR/ACR-2019 appear clinically justified.

## Introduction

Systemic lupus erythematosus (SLE) is a multisystem autoimmune disease that is characterized by the presence of autoantibodies against nuclear antigens, immune complex deposits, and chronic inflammation of typical target organs (1). The complement system plays multiple roles in the pathogenesis of SLE, such as clearing immune complexes, promoting inflammatory responses, eliminating apoptotic cells, and regulating B-cell activation for autoantibody production (2). Low complement levels are a hallmark of SLE and affect disease classification (3).

No clinically established diagnostic criteria are available for SLE, and the diagnosis largely depends on the expertise of senior rheumatologists (4, 5). However, classification criteria for SLE, which are primarily designed to define homogeneous groups for epidemiologic studies and clinical trials, help clinicians determine whether patients meet the clinical and serologic features required for SLE diagnosis (1, 6). International organizations have successively proposed several classification criteria, including the American College of Rheumatology (ACR)-1997, Systemic Lupus International Collaborating Clinics (SLICC)-2012, European League Against Rheumatism and American College of Rheumatology (EULAR/ACR)-2019, and the recently developed SLE Risk Probability Index (SLERPI) (7–10). Significantly different from ACR-1997, all three newer classification criteria consistently included low complement items into their immunology domains and demonstrated improved classification sensitivity (4, 11, 12).

Serum complement levels in patients with SLE are categorized into two groups: normal serum complement (N-com) levels and hypocomplementemia (H-com), with the majority of cases presenting with H-com (13, 14). The most appropriate classification criteria need to be selected to help clinically recognize patients with SLE with varying complement levels, which may reduce the number of missing and delayed diagnoses in such patients and help prevent inappropriate treatment and disease progression. A study from Japan compared the sensitivity of SLICC-2012 and EULAR/ACR-2019 among patients with SLE with N-com and H-com; however, the sample sizes were relatively small, and the effect of excluding the low complement item on sensitivity remains unexplored (15). Therefore, the role of the complement item in the classification criteria for SLE has so far remained understudied. The present study aimed to investigate the role of the low complement item on the performance of SLICC-2012, EULAR/ACR-2019, and the recently proposed SLERPI in an established Chinese cohort (5). We further explored the plausibility that low C3 and low C4 levels have more weighted scores than low C3 or low C4 levels in the EULAR/ACR-2019 classification criteria.

## Methods

### Participants and the study design

The established Chinese cohort included 352 patients with SLE and 385 patients without SLE who were hospitalized in the Department of Rheumatology and Immunology of the Second Affiliated Hospital of Soochow University from January 2016 to December 2020. Rheumatologists with ≥8 years of clinical experience identified all disease diagnoses. The SLE group, excluding those concurrent with other connective tissue diseases (CTDs), infections, cirrhosis, malignancies, or pregnancy, was stratified into SLE groups with H-com (*n* = 312) and N-com (*n* = 40) based on the serum complement level, including C3 and C4 levels. The SLE group with H-com was further categorized into the SLE group with low C3/C4 (*n* = 77) and low C3 and C4 (*n* = 235). The non-SLE group is a general cohort of antinuclear antibodies (ANA)-positive patients with other rheumatic/non-rheumatic diseases (median age 55 (16–83) years, female 72.5%) (4, 5), including patients with immune thrombocytopenic purpura (*n* = 3), liver cancer (*n* = 5), ulcerative colitis (*n* = 7), Crohn’s disease (*n* = 8), urinary tract infection (*n* = 10), lymphoma (*n* = 10), pneumonia (*n* = 11), lung cancer (*n* = 16), Behcet’s disease (*n* = 12), mixed CTD (*n* = 14), spondyloarthritis (*n* = 18), systemic sclerosis (*n* = 24), idiopathic inflammatory myopathies (*n* = 24), primary systemic vasculitis (*n* = 25), rheumatoid polymyalgia (*n* = 25), undifferentiated CTD (UCTD) (*n* = 32), primary Sjogren’s syndrome (pSS) (*n* = 56) and rheumatoid arthritis (*n* = 85).

### Data collection

Patient data were collected from electronic medical records and laboratory results, including gender, age, disease duration, and clinical and immunological items enrolled in the classification criteria. Notably, data were intentionally excluded if there was a more likely explanation than SLE. Subsequently, another staff member reviewed all the collected data before the analysis. Any doubts would be reconfirmed by the two clinical experts to prevent attribution errors where possible. In our hospital immunology laboratory, serum C3 and C4 levels were measured with an immunoscattering turbidimetry assay with H-com defined as C3 of <0.73 g/L and C4 of <0.11 g/L (16), and the ANA titer was measured with an immunofluorescence antibody assay with positive ANA defined as titer of ≥1:100. The classification time was determined through retrospective review of sequential clinical records, where data collectors systematically assessed each visit against classification criteria using electronic health records, documenting the duration from the first occurrence of one item to reaching the threshold specified in the classification criteria. Similarly, the diagnosis time spanned from the first appearance of SLE-related symptoms or abnormal laboratory results to clinical diagnosis by rheumatologists (4, 5).

### Statistical analysis

Categorical variables were presented as absolute frequencies and percentages, using the χ^2^ test or Fisher’s exact test to assess between-group differences. Continuous data were expressed as the median (25th and 75th), using the Mann–Whitney U test for between-group analyses. Statistical Package for the Social Sciences Statistics version 23 (IBM Corp., Armonk, NY, United States) was used for statistical analyses. P (CA, United States) was employed to make the figures. A two-sided *p*-value of <0.05 indicated statistical significance.

## Result

### Clinical and immunological characteristics of patients with SLE with H-com and N-com

The SLE group with H-com demonstrated a significantly younger age distribution compared with the SLE group with N-com (Table 1). No significant differences were observed between the two groups on clinical items, whereas the SLE group with H-com demonstrated a significantly higher prevalence of anti-dsDNA antibody positivity on immunology items based on the classification standards (Table 1). Notably, the total scores of the SLE group with H-com remained significantly higher than the SLE group with N-com in both the SCLCC-2012 and SLERPI, even after excluding the low complement item from the classification criteria (Table 1).

**Table 1:** Clinical characteristics of patients with SLE with normal and low serum complement levels.

Characteristics	SLE with H-com (*n* = 312)	SLE with N-com (*n* = 40)	*P*-value
Age, year	35 (26, 48)	45 (30, 55)	0.024
Female (%)	288 (92.3)	34 (85.0)	0.209
Duration, month	75.5 (24.0, 126.8)	94.5 (27.0, 174.0)	0.287
Total scores of items in the SLICC-2012			
Including low complement	7 (6, 8)	5 (4, 6)	<0.001
Excluding low complement	6 (5, 7)	5 (4, 6)	0.010
Total scores of items in the EULAR/ACR-2019			
Including low complement	22 (18, 28)	17 (14, 21)	<0.001
Excluding low complement	18 (14, 24)	17 (14, 21)	0.166
Total scores of items in the SLERPI			
Including low complement	15.5 (12.0, 18.5)	11.8 (10.5, 13.9)	<0.001
Excluding low complement	13.5 (10.5, 17.0)	11.8 (10.5, 13.9)	0.009
Clinical items, *n* (%)			
Acute cutaneous lupus	150 (48.1)	17 (42.5)	0.506
Chronic cutaneous lupus	2 (0.6)	0 (0)	1.000
Oral ulcer	55 (17.6)	7 (17.5)	0.984
Nonscarring alopecia	118 (37.8)	14 (35.0)	0.729
Synovitis (2 or more joins)	159 (51.0)	23 (57.5)	0.436
Serositis	67 (21.5)	11 (27.5)	0.388
Renal disorder	156 (50.0)	15 (37.5)	0.136
Neurological disorder	15 (4.8)	0 (0)	0.317
Hemolytic anemia	93 (29.8)	7 (17.5)	0.104
Leukopenia (<4,000/mm^3^)	238 (76.3)	27 (67.5)	0.225
Thrombocytopenia (<100,000/mm^3^)	107 (34.3)	8 (20.0)	0.070
Immunology items, *n* (%)			
ANA positivity	310 (99.4)	39 (97.5)	0.304
Anti-dsDNA antibody	225 (72.1)	22 (55.0)	0.026
Anti-Sm antibody	115 (36.9)	13 (32.5)	0.590
The antiphospholipid antibody	44 (14.1)	3 (7.5)	0.248
Low C3/C4	77 (24.7)		
Low C3 and C4	235 (75.3)		
Direct Coombs’ test	26 (8.3)	3 (7.5)	1.000

C3, complement 3; C4, complement 4; H-com, less than expected serum levels of one or more complements in C3 and C4; N-com, normal serum C3 and C4 levels; ACR-1997, 1997 American College of Rheumatology classification criteria; SLICC-2012, 2012 Systemic Lupus International Collaborating Clinics classification criteria; EULAR/ACR-2019, 2019 European League Against Rheumatism/American College of Rheumatology classification criteria; SLERPI, Systemic Lupus Erythematosus Risk Probability Index.

### Effect of low complement item on the performance of different SLE classification criteria

**Figure 1:**
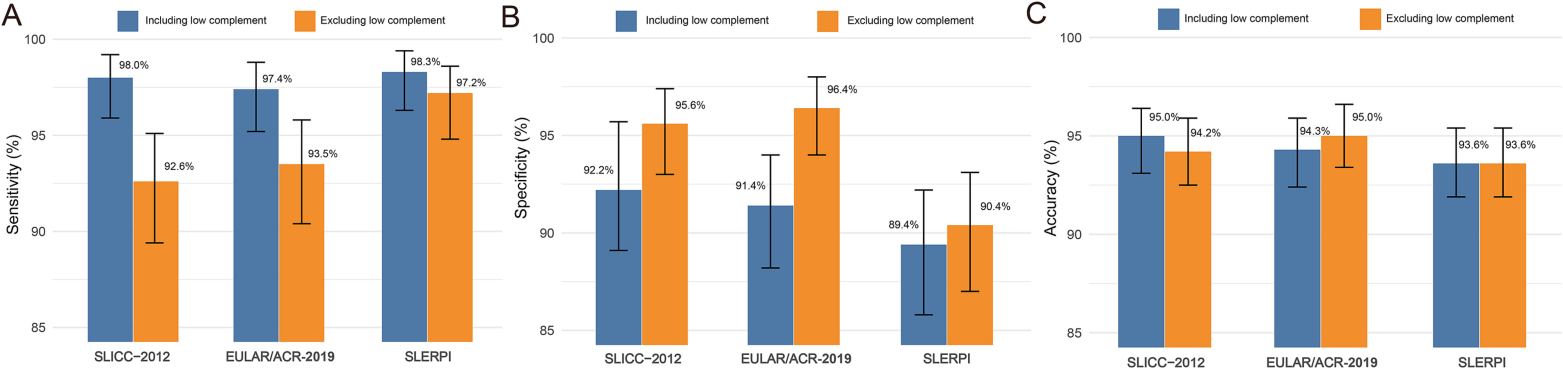
The performance of different SLE classification criteria. The sensitivity **(A)**, specificity **(B)**, and accuracy **(C)** of different SLE classification criteria before and after excluding low complement.

The classification performance evaluation revealed distinct characteristics among the three classification criteria. SLERPI demonstrated superior sensitivity (98.3%; 95% confidence interval [CI]: 96.3–99.4%), whereas SLICC-2012 exhibited the highest specificity (92.2%; 95% CI: 89.1–95.7%) (Figure 1). After excluding the low complement item, SLICC-2012 and EULAR/ACR-2019 showed decreased sensitivity and increased specificity but little change in accuracy. Intriguingly, SLERPI demonstrated minimal fluctuations in diagnostic sensitivity, specificity, and accuracy compared with SLICC-2012 and EULAR/ACR-2019 after removing low complement from the criteria.

### Effect of low complement item on the sensitivity of different classification criteria for patients with SLE with H-com and N-com

SLICC-2012 exhibited different classification sensitivities for patients with SLE with different complement status, with a sensitivity of 99.0% (95% CI: 97.2–99.8%) and 90.0% (95% CI: 76.3–97.2%) in patients with H-com and N-com, respectively. Further, the classification sensitivity of EULAR/ACR-2019 was slightly higher in patients with H-com (97.7%; 95% CI: 95.4–99.1%) than in those with N-com (95.0%; 95% CI: 83.1–99.4%). However, the classification sensitivity of SLERPI (98.4%; 95% CI: 96.3–99.5% vs. 97.5%; 95% CI: 86.8–99.9%) exhibited minimal variation between the two groups (Table 2). Therefore, the status of complement in patients with SLE affected the classification sensitivity of SLICC-2012 and EULAR/ACR-2019, but to a lesser extent, SLERPI.

**Table 2:** Sensitivity of different SLE classification criteria in identifying SLE with H-com and N-com after excluding low complement.

Classification criteria	Low complement status	Sensitivity (95%CI), %	
		SLE with H-com	SLE with N-com
SLICC-2012	Including low complement	99.0 (97.2, 99.8)	90.0 (76.3, 97.2)
	Excluding low complement	93.0 (89.5, 95.5)	–
EULAR/ACR-2019	Including low complement	97.7 (95.4, 99.1)	95.0 (83.1, 99.4)
	Excluding low complement	93.3 (90.0, 95.8)	–
SLERPI	Including low complement	98.4 (96.3, 99.5)	97.5 (86.8, 99.9)
	Excluding low complement	97.1 (94.6, 98.7)	–

We then explored the effect of the low complement item on the classification sensitivity of different classification criteria for patients with SLE with H-com. After excluding the low complement item, the classification sensitivity of SLICC-2012 and EULAR/ACR-2019 for patients with H-com was reduced to 93.0% (95% CI: 89.5–95.5%) and 93.3% (95% CI: 90.0–95.8%), respectively (Table 2). In contrast, SLERPI (97.1%; 95% CI: 94.6–98.7%) maintained a stable sensitivity.

### Impact of low complement item on the early identification of patients with SLE with H-com

Our previous study demonstrated that SLERPI performed well in the early identification of SLE (4); therefore, we further assessed the association of the low complement item with the early identification of patients with SLE with H-com. The time of clinical diagnosis was unclear in 45 patients with SLE with H-com; thus, we investigated it in the remaining 267 patients with a precise time of clinical diagnosis. The results indicated that SLICC-2012, EULAR/ACR-2019, and SLERPI identified 16, 17, and 21 patients with SLE with H-com earlier than the clinical diagnosis, respectively. The exclusion of the low complement item reduced the number of patients with SLE identified early in SLICC-2012 by six, whereas the number of those with SLE identified early in EULAR/ACR-2019 and SLERPI was reduced by only one (Table 3).

**Table 3:** Classification time of different SLE classification criteria after excluding low complement compared with clinical diagnosis time.

Classification criteria	Low complement status	Earlier	At one time	Later	*N*
SLICC-2012	Including low complement	16	233	15	264
	Excluding low complement	10	223	19	252
EULAR/ACR-2019	Including low complement	17	237	6	260
	Excluding low complement	16	229	8	253
SLERPI	Including low complement	21	237	6	264
	Excluding low complement	20	233	8	261

### Low C3 and low C4 deserve more weighted scores in the EULAR/ACR-2019 classification criteria

The EULAR/ACR-2019 classification criteria assigned low C3 or low C4 with 3 weighted scores, whereas low C3 and low C4 are assigned 4 weighted scores. Therefore, we finally investigated the plausibility that low C3 and low C4 levels have more weighted scores than low C3 or low C4 levels. The results revealed that patients with SLE with low C3 and C4 levels demonstrated a markedly shorter disease duration compared with those with SLE with low C3 or C4 levels (Table 4). Regarding the clinical items included in the classification criteria, acute cutaneous lupus and nonscarring alopecia manifestations were significantly more prevalent in patients with SLE with low C3 and low C4 levels. In the immunology items, positive anti-DNA antibodies, antiphospholipid antibodies, and direct Coombs’ tests were more prevalent, and the total scores were significantly higher in the SLE group with low C3 and C4 than in the SLE group with low C3 or C4 levels in all three classification criteria, SLICC-2012, EULAR/ACR-2019, and SLERPI. This significance persisted even after excluding the low complement item (Table 4).

**Table 4:** Clinical characteristics of patients with SLE with low C3/C4 and low C3 and C4 levels.

Characteristics	SLE with low C3/C4 (*n* = 77)	SLE with low C3 and C4 (*n* = 235)	*P*-value
Age, year	37 (26, 49.5)	33 (26, 33)	0.333
Female (%)	72 (93.5)	216 (91.9)	0.649
Duration, month	89.0 (56.5, 168.0)	65.0 (12.0, 120.0)	0.001
Total scores of items in the SLICC-2012			
Including low complement	6 (5, 7)	7 (6, 8)	<0.001
Excluding low complement	5 (4, 6)	6 (5, 7)	<0.001
Total scores of items in the EULAR/ACA −2019			
Including low complement	18 (14, 24)	23 (19, 28)	<0.001
Excluding low complement	15 (11, 21)	19 (15, 24)	<0.001
Total scores of items in the SLERPI			
Including low complement	11.50 (9.00, 16.25)	16.50 (13.50, 19.50)	<0.001
Excluding low complement	11.50 (9.00, 16.25)	14.50 (11.50, 17.50)	<0.001
Clinical items, *n* (%)			
Acute cutaneous lupus	28 (36.4)	122 (51.9)	0.018
Chronic cutaneous lupus	0 (0)	2 (0.9)	1.000
Oral ulcer	9 (11.7)	46 (19.6)	0.115
Nonscarring alopecia	16 (20.8)	102 (43.4)	<0.001
Synovitis (2 or more joins)	38 (49.4)	121 (51.5)	0.745
Serositis	15 (19.5)	52 (22.1)	0.623
Renal disorder	36 (46.8)	120 (51.1)	0.511
Neurological disorder	2 (2.6)	13 (5.5)	0.461
Hemolytic anemia	19 (24.7)	74 (31.5)	0.257
Leukopenia (<4,000/mm^3^)	57 (74.0)	181 (77.0)	0.592
Thrombocytopenia (<100,000/mm^3^)	24 (31.2)	83 (35.3)	0.506
Immunology items, *n* (%)			
ANA positivity	77 (100.0)	233 (99.1)	1.000
Anti-dsDNA antibody	44 (57.1)	181 (77.0)	0.001
Anti-Sm antibody	26 (33.8)	89 (37.9)	0.517
The antiphospholipid antibody	3 (3.9)	41 (17.4)	0.003
Direct Coombs’ test	2 (2.6)	24 (10.2)	0.036

## Discussion

All three classification criteria, SLICC-2012, EULAR/ACR-2019, and SLERPI, coincidentally include the low complement item and assign different values. We revealed that the low complement item demonstrated the most significant effect on the sensitivity and early identification ability of SLICC-2012 and the least effect on SLERPI. Further, clinical and serologic activity was more frequent in patients with SLE with both low C3 and C4 levels, which supports the rationale for a higher weighting of low C3 and C4 levels in the EULAR/ACR classification criteria. The complement system can be activated with the classical, alternative, and lectin pathways involved in SLE’s pathogenesis and disease activity. The formation of immune complexes may generally cause complement depletion. Swaak et al. (17) demonstrated that anti-dsDNA antibodies that are specific for SLE bind to DNA to activate the complement system, resulting in decreased C3 and C4. Further, complement activation has driven anti-dsDNA antibody development (18). Thus, complement-related SLE studies revealed a significantly higher anti-dsDNA antibody positivity rate in the SLE group with H-com than in the SLE group with N-com (15, 19), which is consistent with our described results.

Consistent with the previous study conducted in Colombia (20), we revealed that SLERPI exhibited a higher sensitivity than SLICC-2012 and EULAR/ACR-2019 for identifying SLE. After excluding the low complement item, the sensitivity of SLICC-2012 dramatically decreased, whereas the influence on the performance of SLERPI was limited. Similarly, when these three classification criteria were used to identify SLE with H-com, the exclusion of low complement markedly reduced the sensitivity of the SLICC-2012 but slightly decreased the sensitivity of SLERPI. Aringer et al. (9) revealed that the SLICC-2012 criteria demonstrated higher sensitivity than the ACR1997 criteria, with a significant contributing factor being the inclusion of low complement levels. A possible explanation is that SLICC-2012 requires at least one clinical and one immunological criterion for SLE classification; therefore, patients who meet the classification criteria based on immunological low complement alone are no longer identified as patients with SLE when the low complement item is removed from SLICC-2012. Hence, the low complement item has a specific impact on the classification efficacy of SLICC-2012.

Intriguingly, our study demonstrated that the low complement item had little effect on the performance of SLERPI. Among the SLERPI, proteinuria, thrombocytopenia, and hemolytic anemia are clinical predictors with greater weight (8). Previous studies have revealed that low complement levels are usually associated with lupus nephritis presenting as proteinuria (21). Further, Jiang et al. observed that H-com was independently associated with thrombocytopenia (22). In our study, the other high-weighted SLERPI items like thrombocytopenia (34.3% vs. 20%), renal disorder (50% vs. 37.5%), and anti-dsDNA (72.1% vs. 55%) were more prevalent in H-com vs. N-com groups. Thus, we hypothesized that SLE with H-com would have highly weighted clinical or serologic manifestations, and they would still meet the scoring requirements when scored with SLERPI even if the low complement item was not considered. Furthermore, SLICC-2012 included low C3 or C4 or CH50, EULAR/ACR-2019 included low C3 or C4/low C3 and C4, whereas SLEPRI included only low C3 and C4 (8–10). The majority of our enrolled patients with SLE with H-com demonstrated a combination of low C3 and low C4. The following analysis revealed that patients with SLE with low C3 and C4 were more likely than patients with C3/C4 to have acute cutaneous lupus, proteinuria, positive anti-dsDNA antibodies, or positive antiphospholipid antibodies, all of which were included in the SLERPI scoring system. Thus, these patients could easily score above 7 and be classified as having SLE, even excluding the low complement item from the SLERPI. SLERPI aimed to propose a simple, clinician-friendly classification criterion to assist in identifying SLE (8). Hence, removing the low complement item would make SLERPI a more concise classification criterion.

The current study further compared the sensitivity of patients with SLE with different complement statuses. The sensitivity to determine SLE with H-com was highest and significantly higher than SLE with N-com for SLICC-2012; whereas, it was moderately higher than SLE with N-com for EULAR/ACR-2019, and was slightly higher than SLE with N-com for SLERPI. Previous research has revealed a significant correlation between low complement and meeting SLICC-2012 and between concomitant low C3 and C4 levels and meeting EULAR/ACR-2019 (23). These results indicate that the inclusion of low complement in the domains was significantly associated with the performance of SLICC-2012 and EULAR/ACR-2019 in identifying SLE with H-com. These findings suggest that rheumatologists should adopt a complement status-driven approach to SLE classification in clinical practice. For SLE with H-com, SLICC-2012 is preferentially recommended, while for SLE with N-com, the SLERPI is more appropriate‌.

Early SLE diagnosis is essential to initiate timely treatment, which can increase the likelihood of remission and improve patient prognosis (24). Our previous study revealed that SLERPI enabled earlier classification of patients with SLE compared with the clinical diagnosis and EULAR/ACR-2019 criteria (4). Studies have demonstrated that low C3/C4 was more predominant in severe SLE following the Lupus Severity Index score, and SLE disease activity was higher in SLE with H-com based on the SLE Disease Activity Index (19, 25). Therefore, early identification of patients with SLE with H-com is crucial to facilitate early treatment and prevent disease progression and activity as much as possible. In our current study, SLERPI’s ability to identify SLE with H-com earlier remained better than SLICC-2012 and EULAR/ACR-2019, and was not significantly weaker after excluding low complement. This indicates that SLERPI is more recommended for the earlier clinical identification of SLE in patients with CTDs with low complement.

The EULAR/ACR-2019 classification criteria assigned greater weight to cases with both low C3 and C4 levels. In our study, patients with SLE with both low C3 and C4 levels demonstrated shorter disease duration and increased frequency of clinical and immunologic manifestations compared with those with isolated low C3 or C4 levels. Duncan et al. investigated the associations between isolated low C3, isolated low C4, and combined low C3 and C4 with clinical and serological profiles in patients with SLE. Isolated low C3 was correlated with anti-dsDNA and anti-Sm autoantibodies, renal involvement, and hematologic manifestations. Isolated low C4 was associated with anti-dsDNA, anticardiolipin antibodies, and false-positive rapid plasma tests. Notably, a combination of low C3 and C4 levels was strongly associated with serositis, renal injury, seizures, and all serologic and immunological abnormalities (14). Another study exploring the associations between different low complement statuses and clinical/laboratory parameters in an adolescent SLE cohort found a comparable correlation (26). Further, a study demonstrated that the progressive reduction in C3 and C4 levels was significantly related to higher relapse rates (16). These results indicate that patients with SLE with low C3 and C4 levels exhibited broader clinical manifestations and pronounced immune activation. Therefore, assigning a higher weight to both low C3 and C4 levels is reasonable, which could improve the sensitivity and accuracy of the SLE classification criteria and facilitate earlier identification of these patients.

This study has several inherent limitations that should be acknowledged. The single-center design with a modest sample size and exclusive recruitment of Han Chinese patients may limit the generalizability of our findings, given known ethnic variations in complement system activation and disease manifestations across populations. Moreover, the retrospective nature of data collection, relying on electronic medical records for classification and diagnosis timelines, may introduce potential information bias. In addition, the absence of longitudinal complement monitoring is a significant limitation, as complement levels can fluctuate and influence classification differently over time. These findings warrant validation through future multicenter prospective studies incorporating ethnically diverse cohorts and standardized complement profiling methodologies to enhance clinical applicability.

## Conclusion

Our analysis revealed that the inclusion of the low complement item strongly improved the sensitivity and early detection capacity of SLICC-2012 while minimally influencing SLERPI. Further, EULAR/ACR-2019 reasonably assigns higher weighted scores to combined low C3 and C4, which helps in clinical application. However, these conclusions should be interpreted with caution, and prospective validation in multicenter studies is warranted.

## Data Availability

The raw data supporting the conclusions of this article will be made available by the authors, without undue reservation.
